# Family support and transport cost: understanding health service among older people from the perspective of social-ecological model

**DOI:** 10.1186/s13690-022-00923-1

**Published:** 2022-07-19

**Authors:** Bocong Yuan, Tong Zhang, Jiannan Li

**Affiliations:** 1 School of Tourism Management, Sun Yat-sen University, West Xingang Rd. 135, Guangzhou, China; 2 Institute of Advanced Studies in Humanities and Social Sciences, Beijing Normal University, Jinfeng Rd. 18, Zhuhai, 519087 China

**Keywords:** Health care service, Family support, Transport, Disability, Older people

## Abstract

**Background:**

This study is to investigate the interaction of family support, transport cost (ex-post), and disabilities on health service seeking behavior among older people from the perspective of social ecological model.

**Method:**

We conduct a series of regressions including the Poisson model and Multiple logit model. The Heckman two-stage procedure is also conducted to check the robustness.

**Results:**

Given that health care resources are generally concentrated in densely populated urban areas, access to services of higher-class health care facilities is found associated with higher transport cost (ex-post). Family support could also promote the access to higher-class health care facilities. Although disability may impede such access, this effect may be mitigated by paying higher transport cost (ex-post).

**Conclusions:**

Alleviating transport deprivation and promoting family support are critical for access to better healthcare services among older people with disabilities.

## Background

Availability, affordability, and acceptability are three pillars of equity in health care access. Health care service is critical for older people, as they are more prone to chronic conditions such as cardiovascular disease, physical illness, disability and cognitive impairment, and mental health problems [1, 2]. Differences in travel conditions (e.g., distance, time, and expenditure) are important for their decision-making of health care utilization [3, 4]. The role of transport conditions between residence and health facilities in access to health services is more outstanding in low- and middle-income countries (LMICs) and for people with disabilities [5, 6]. The health inequity remains prevalent among older people with obstacles in accessing health care services, exacerbating their risk of underdiagnosis [7]. The barriers to health care facilities reduce their utilization of preventive health services and treatments [8, 9]. In practice, the political marginalization, discrimination, and unequal access to health care services faced by people with disabilities lead to their worse health status than their counterparts [10, 11].

Besides, social/family support is another of the most proximal determinants of access to health services for older people with disabilities [12], especially in the LMICs context where people often face resource constraint in decision making. Though the effect of social support on health behaviors and decisions about health care seeking is recognized [13], the research on the role of family support, as a more proximal source of care for older people, is insufficient [14]. Decisions about health care services seeking are made against the context of social networks that are influenced by family members and peer groups in ways such as financial support [15]. Older people who receive sound support and contact with their children may have a stronger sense of social role, promoting their willingness to seek active medical treatment.

According to the social ecological model, the behavioral decision is often subjected to different levels of the environment (i.e., individual, interpersonal, organizational, community and policy) [16], and the decision-making of health service seeking is conditioned on the environment where people live in. By extending the social ecological model into the medical treatment issue of older people with disabilities, this study could expand the development of social ecological model and enrich the understanding of this topic. In this study, disabilities, family support and transport will be integrated into the decision making of health care service seeking among this vulnerable group based on this theoretical framework.

## Literature and hypotheses

### Family support and access to health care service in the perspective of social ecological model

The social ecological model provides a theoretical framework for explaining older people’s decision-making about access to health care services by dividing external determinants into micro and macro levels [17]. Specifically, the individual level (e.g., personal characteristics like gender, education, health status, etc.), the interpersonal level (e.g., social interaction of family members and friends), the organizational level (e.g., activities carried out by organizations like advices and professional counselling), the community level (e.g., social, and cultural aspects of living environments), and policy level (e.g., governmental regulations and initiatives) constitute determinants from proximal to distal [16]. In this study, from the perspective of social ecological modeling, disability serves as an individual characteristic, while family support and transportation correspond to interpersonal and community level.

In practice, family support is considered as positive attitudes, behaviors, and family acceptance [18]. Interpersonal interactions with families can be an important channel to connect oneself to social networks that is important for one’s health and behavioral decisions [19], especially for older adults retiring from the workforce. Family care creates a sense of belonging, self-esteem, and positive self-perception, family encouragement and assistance will enable people to take care of their health and enhance their willingness to use remote services [20]. Likewise, emotional and practical support from family members can reduce the occurrence of psychological distress and thus lower the depression levels [21, 22].

Moreover, the support from families can be broadly divided into financial and non-financial support. Studies have shown that the support from children is positively correlated with the tendency of older patients in rural areas to seek health services [23]. Older people in retirement will face reduced income, thus financial conditions become a major barrier to access to better health care with lower affordability. In their daily life, older adults could benefit from basic care provided by family members, which helps them maintain good health [24]. Besides, family support provides emotional connections that help them maintain greater efficacy in seeking health services [25]. Sympathy and care of family members can provide emotional support for older people with reduced social activities, leading to higher levels of mental health. Therefore, we propose

- *Hypothesis 1. Greater family support is associated with the visit to higher-class facilities among older people*

### Transport and access to health service

Limited availability of health care facilities within reach implies that high out-of-pocket expenditures, long distances to health care facilities and high cost of travel time are the most important impediments to the access to health care [26]. Travel conditions are important for patients with chronic diseases that require multiple follow-up visits [27]. Difficulties or obstacles in reaching health care facility may reduce people’s use of medication [28]. For example, people with diabetes suffer from inadequate insulin use [29] and poor blood glucose control [30] due to long driving distances from home to health care providers. Patients with mental health problems who are unable to drive believe that transportation can significantly affect their accessibility to health facilities and limit their medical compliance [31], leading to the reduction in their drug supplementation [32].

In prior studies, transport cost can be manifested as longer travel distance or longer travel time. In many developing countries, there is an association between distance and access to health services [33], reflecting lower frequency of utilization of health services [34]. In general, advanced health care facilities are located in densely populated urban areas, thus older people, especially older rural residents, face greater transportation barriers to access to health care facilities compared with their counterparts [35, 36]. Longer travel distances could lead to the longer time to reach health care facilities and thus the longer time in the health services seeking [3]. As such, it is often observed that the ex-post longer transport distance is associated with the visit to advanced medical facilities, which would not otherwise be considered worthwhile.

Although travel time and distance are thought to be important determinants of people’s access to health care, the mechanisms may not be intuitive. Lack of personal resources can be a critical deterrence of reaching health service facilities and makes necessary care less affordable especially in LMICs where non-motorized rickshaw and vans are prevalent [37]. Given similar distance, different modes of transportation can also lead to differences in travel time, resulting in the disadvantage in utilization of health services [38, 39]. Trips to medical services may be time-consuming because older people cannot drive on their own and public transport options are often limited [40]. Thus, we propose

- *Hypothesis 2. The ex-post transport cost is positively associated with the visit to higher-class facilities among older people*

### Disability and access to health service

Older people with disabilities have a greater need for health care services (e.g., protective services) and face greater barriers to access to health care than their counterparts [41]. Older people are often faced with different types of disabilities (e.g., movement restriction, cognitive impairment, hearing, and vision impairment) [42]. But only a small part of the disabled have their health care needs met [43]. The research on African Americans showed that inaccessible or inconvenient transportation was an important impediment to health service seeking for people with mobility disabilities [44]. Due to the imperfect transportation services, people with movement restriction are often unable to take public transportation to reach health care facilities located in crowded urban areas, but turn to nearby traditional doctors for treatment [45]. For those with hearing impairments, the difficulty in communicating is the biggest challenge they face when seeking health services [46]. The barriers to access to health-care facilities can be categorized as the financial, structural, and procedural for older people with disabilities, which include the cost of travel to health care facilities, the long distance to health care facilities, lack of companions, language and communication problems, and staff attitudes, etc. [47]. Some of these challenges are shared by older people with and without disabilities. People with disabilities may face greater disadvantages in terms of information and geographical accessibility when accessing medical facilities [48]. Disability may exacerbate difficulties in accessing higher-level health care facilities located in crowded urban areas. Thus, we propose

- *Hypothesis 3. Disability is negatively associated with the class of health care service facilities visited among older people*
- *Hypothesis 4. The negative relationship between disability and the class of health care service facilities visited among older people can be alleviated by paying higher transport cost*

## Material and method

### Description of data

This study applied the data from China Health and Retirement Longitudinal Study (CHARLS-2015 wave, and the harmonized version) which is initiated for the purpose of tracking health status and their health care service utilization among older population. This survey is among the very limited data source that tracks both the family support and utilization of transport in the process of health care service seeking among older people with different disabilities. The survey involves a nationally representative sample that covers about 150 counties (450 communities/villages nationwide) in China, among which 47.4% are of urban areas. This study is exempted from reviewing by institutional review board for the application of publicly available data and authors have no contact with the respondents.

### Variables

#### Dependent variables

*Class of health care facilities visited* is measured using two methods for robustness. Firstly, it is coded =7 general hospital; =6 specialized hospital; =5 Chinese medicine hospital; =4 community healthcare center; =3 township hospital; =2 health care service station; =1 village clinic/private clinic. Secondly, it is coded according to the three-tier health system. For outpatient service, coded =3 general hospital; =2 specialized hospital, Chinese medicine hospital; =1 community healthcare center, township hospital, health care service station, village clinic/private clinic.

#### Independent variables

*Disabilities* is measured by the number of types of following problems he/she suffers (physical disabilities, brain damage/mental retardation, vision problem, hearing problem, speech impediment).

*Transport cost in health care seeking (ex-post, having already paid)* is measured by the distance from home to the health care facility most recently visited (km, one-way).

*Family support* is measured using two methods for robustness (i.e., face-to-face and online connection). The former is measured by inquiring “How often would you/your spouse see your child who is not living with you?” The latter is measured by inquiring “How often would you/your spouse contact your child who is not living with you on phone/by message/on WeChat/by mail/by email?” (higher score implies greater family support, from 1 to 10). This variable is coded according to the mean value of his/her rating about all children if he/she has more than one child.

#### Covariates

*Demographics* including age, gender, the beneficiary status of health insurance, financial status, chronic condition, and self-rated health. More details about description of variables are displayed in Table 1.

**Table 1:** Description of variables

Variables	Description	Details	Freq.	%
*Class of health care facilities visited*	Measured in two types. Firstly, =7 general hospital; =6 specialized hospital; =5 Chinese medicine hospital; =4 community healthcare center; =3 township hospital; =2 health care service station; =1 village clinic/private clinic. Secondly, in the robustness check section, it would also be classified according to the three-tier health system. For outpatient service, =3 general hospital; =2 specialized hospital, Chinese medicine hospital; =1 community healthcare center, township hospital, health care service station, village clinic/private clinic.	*Village clinic/Private clinic (first tier)*	346	18.82
		*Health care service station (first tier)*	40	2.18
		*Township hospital (first tier)*	347	18.88
		*Community healthcare centers (first tier)*	106	5.77
		*Chinese medicine hospital (second tier)*	119	6.47
		*Specialized hospital (second tier)*	88	4.79
		*General hospital (third tier)*	792	43.09
*Transport cost in health service seeking [ex-post, having paid]*	Measured by the distance from home to the health care facility most recently visited (km). It reflects the transport cost that the respondent has already paid in the visit he/she most recently completed. In regressions, it is calculated in natural log (i.e., *ln [1 + transport cost]*).	*< 5*	1015	55.22
		*5-10*	339	18.45
		*11-20*	189	10.28
		*21-30*	98	5.34
		*> 30*	194	10.56
*Disability*	The number of types of following problems suffered (physical disabilities, brain damage/mental retardation, vision problem, hearing problem, speech impediment)	*0*	1581	86.02
		*1*	201	10.94
		*2*	43	2.34
		*3*	11	0.60
		*4*	2	0.11
*Family support (face to face)*	How often would you/your spouse see your child who is not living with you? (=10 almost every day, =9 if 2-3 times a week, =8 once a week, =7 every two weeks, =6 once a month, =5 once every three months, =4 once every six month, =3 once a year, =2 almost never, =1 others). The research team inquire about the situation of all children of the respondent. This variable is coded according to the mean value of his/her rating about all children.	*[1, 8]*	173	9.41
		*(8, 9]*	356	19.37
		*(9, 10]*	1309	71.21
*Family support (online connection)*	Coded similar as above, but the question is “How often would you/your spouse contact your child who is not living with you on phone/by message/on WeChat/by mail/by email?”	*[1, 8]*	85	4.62
		*(8, 9]*	199	10.83
		*(9, 10]*	1554	84.55
*Age*	The year of age.	*< 55*	667	36.29
		*55-65*	641	34.87
		*66-75*	398	21.66
		*> 75*	132	7.17
*Gender*	=1 male, =2 female.	*1. male*	741	40.32
		*2. female*	1097	59.68
*Health insurance*	=1 if the respondent has at least one of the following social health insurances: urban employee basic medical insurance (UEBMI), urban resident basic medical insurance (URBMI), new cooperative medical scheme (NCMS), urban and rural resident medical insurance (URRBMI).	*No*	77	4.19
		*Yes*	1761	95.81
*Financial status*	The amount of cash a respondent has (in natural log)	*< 3.00*	303	16.49
		*3.00-7.00*	929	50.54
		*7.00-10.00*	578	31.45
		*10.00-13.00*	28	1.52
*Chronic conditions*	The number of types of following chronic conditions suffered, including hypertension; dyslipidemia; diabetes or high blood sugar; cancer or malignant tumor; chronic lung diseases; liver disease; heart attack, coronary heart disease, angina, congestive heart failure, or other heart problem; stroke; kidney disease; stomach or other digestive disease; emotional, nervous, or psychiatric problems; memory-related disease; arthritis or rheumatism; asthma.	*0*	813	44.23
		*1*	567	30.85
		*2*	258	14.04
		*3*	99	5.39
		*4*	63	3.43
		*5*	28	1.52
		*6*	7	0.38
		*7*	2	0.11
		*8*	0	0.00
		*9*	1	0.05
*Self-rated health*	Self-reported general health status. =1 very good, … =5 very poor.	*1.Very good*	99	5.39
		*2.Ggood*	160	8.71
		*3. Fair*	940	51.14
		*4.Poor*	*494*	26.88
		*5.Very poor*	145	7.89

The descriptive statistics are conducted based on the sample observations in which data of all variables are non-missing for outpatient service

### Approach

The Poisson regressions adjusted with robust standard errors on individual level are conducted to examine the influences of transport cost of health service seeking, family support and disability on the class of health care facilities. There are two reasons for using this method. First, the Poisson regression is suitable for the situation where the dependent variable is valued with non-negative integers. Second, since individuals can show non-randomized variations in income, insurance, etc., the violation of independent and identical distribution can bring bias in the regression analysis. The application of robust standard errors for adjustment in the regression analysis is to overcome the weakness of heterogeneity on individual level that could result in estimation bias.

In the robustness check section where the dependent variable is coded according to the three-tier system, the multiple logit model is applied, as the dependent variable has three values. The Stata 16.0 (Stata Corp. LLC., College Station, TX, USA) is applied in the analysis. The regression is shown as below.

$$Class\ of\ health\ care\ facilities\ visited=\beta 0+\beta 1\ Transport\ cost\ \left( ex- post\right)+\beta 2\ Disabilities+\beta 3\ Family\ support+\beta 4\ Transport\ cost\ \left( ex- post\right)\times Disabilities+\beta 5\ Age+\beta 6\ Gender+\beta 7\ Education+\beta 8\ Marriage+\beta 9\ Health\ insurance+\beta 10\ Financial\ status+\beta 11\ Chronic\ conditions+\beta 12\ Self- rated\ health+\varepsilon$$

## Empirical results

Table 1 displays that 63.71% of respondents are aged > 55 years. 59.68% are female. 55.22% take ≤5 km on travel between residence place and health care facilities. 44.23% report that they have no chronic condition.

Empirical results (Table 2, Model 1) show that, when measuring family support with the face-to-face type, transport distance between residence place and health care facilities (coefficient = 0.139, 95% CI [0.124, 0.153], *p* < 0.01) and family support (coefficient = 0.048, 95% CI [0.023, 0.073], *p* < 0.01) are both positively associated with the class of health care facilities visited. In contrast, disability status is negatively associated with the class of health care facilities visited (coefficient = − 0.113, 95% CI [− 0.183, − 0.043], *p* < 0.01).

**Table 2:** Results of baseline model (Poisson regression)

	*Dependent variable: the class of health care facility visited [Poisson regression]*					
	*Model 1*			*Model 2*		
	Coef.	Robust S.E.	95% CI	Coef.	Robust S.E.	95% CI
**Independent variables**						
*Transport cost in health service seeking [ex-post, having paid]*	0.139 ^**^	0.007	[0.124, 0.153]	0.139 ^**^	0.007	[0.124, 0.153]
*Family support [face to face]*	0.048 ^**^	0.013	[0.023, 0.073]			
*Family support [Online connection]*				0.071 ^**^	0.018	[0.035, 0.106]
*Disability*	−0.113 ^**^	0.036	[−0.183, −0.043]	−0.114 ^**^	0.035	[−0.184, −0.045]
*Disability ×Transport cost in health service seeking [ex-post, having paid]*	0.033 ^**^	0.011	[0.010, 0.055]	0.035 ^**^	0.011	[0.013, 0.057]
**Demography**						
*Age*	0.003 ^**^	0.001	[0.001, 0.006]	0.004 ^**^	0.001	[0.001, 0.006]
*Gender*						
*Male*	REF.			REF.		
*Female*	0.004	0.021	[−0.037, 0.045]	0.004	0.021	[− 0.037, 0.045]
*Health insurance*	−0.135 ^**^	0.045	[−0.223, − 0.048]	−0.139 ^**^	0.044	[−0.226, − 0.052]
*Financial situations*	0.016 ^**^	0.004	[0.008, 0.023]	0.015 ^**^	0.004	[0.008, 0.022]
*Chronic conditions*	0.025 ^**^	0.008	[0.009, 0.041]	0.024 ^**^	0.008	[0.009, 0.040]
*Self-rated health*	−0.027 ^**^	0.011	[− 0.049, − 0.004]	−0.026	0.011	[−0.049, − 0.004]
Intercept	− 0.139	0.172	[− 0.476, 0.197]	−0.382	0.217	[−0.808, 0.043]
Num. of non-missing observations	1838			1838		
Wald-statistics	530.44			535.41		
[*p*-value]	[0.000]			[0.000]		

^*^p < 0.05, ^**^p < 0.01. Robust standard errors are reported

The above results remain robust when measuring family support with the online connection type instead (Table 2, Model 2). In this case, transport distance between residence place and health care facilities (coefficient = 0.139, 95% CI [0.124, 0.153], *p* < 0.01) and family support (coefficient = 0.071, 95% CI [0.035, 0.106], *p* < 0.01) both remain positively associated with the class of health care facilities visited. In contrast, disability status remains negatively associated with the class of health care facilities visited (coefficient = − 0.114, 95% CI [− 0.184, − 0.045], *p* < 0.01).

Table 3 presents the results of robustness check, where the class of health care facilities visited is categorized according to the three-tier health system (see Table 1 for details). Compared with the baseline group (lowest tier), the transport cost in health service seeking (ex-post) is associated with the visit to higher tier health care facilities (1.022, *p* < 0.01 for the second tier) and (0.966, *p* < 0.01 for the third tier). Family support (face-to-face) is also associated with the visit to higher tier health care facilities (0.332, *p* < 0.01 for the second tier) and (0.274, *p* < 0.01 for the third tier). Disability is negatively associated with the tier health care facilities visited (− 0.882, *p* < 0.05 for the second tier) and (− 0.835, *p* < 0.01 for the third tier). Further, the transport cost in health service seeking (ex-post) might alleviate the negative relationship between disability and the tier health care facilities visited (ex-post) (interaction term = 0.317, *p* < 0.05 for the second tier) and (interaction term = 0.329, *p* < 0.05 for the third tier). These results remain robust when the family support is measured by the online connection instead.

**Table 3:** Supplementary robustness check: Multiple logit model, (three-tier health system)

	Dependent variables: Class of health care facilities visited [multiple logit model, REF = *first tier (community healthcare centers, township hospital, health care service station, village clinic/private clinic)*]											
	*Model 1*						*Model 2*					
	*second tier (specialized hospital, Chinese medicine hospital)*			*third-tier (general hospital)*			*second tier (specialized hospital, Chinese medicine hospital)*			*third-tier (general hospital)*		
	Coef.	Robust S.E.	95% CI	Coef.	Robust S.E.	95% CI	Coef.	Robust S.E.	95% CI	Coef.	Robust S.E.	95% CI
**Independent variables**												
*Transport cost in health service seeking [ex-post, having paid]*	1.022 ^**^	0.079	[0.867, 1.176]	0.966 ^**^	0.065	[0.838, 1.093]	1.022 ^**^	0.079	[0.867, 1.177]	0.971 ^**^	0.066	[0.843, 1.100]
*Family support [face to face]*	0.332 ^**^	0.091	[0.153, 0.510]	0.274 ^**^	0.068	[0.140, 0.408]						
*Family support [Online connection]*							0.342 ^**^	0.124	[0.100, 0.584]	0.404 ^**^	0.097	[0.213, 0.594]
*Disability*	−0.882 ^*^	0.358	[−1.584, −0.180]	−0.835 ^**^	0.271	[−1.366, − 0.303]	− 0.887 ^*^	0.356	[−1.584, − 0.189]	− 0.837 ^**^	0.270	[− 1.366, − 0.307]
*Disability ×Transport cost in health service seeking [ex-post, having paid]*	0.317 ^*^	0.157	[0.009, 0.625]	0.329 ^*^	0.136	[0.063, 0.596]	0.327 ^*^	0.158	[0.017, 0.638]	0.342 ^*^	0.138	[0.072, 0.613]
**Demography**												
*Age*	−0.003	0.009	[− 0.022, 0.015]	0.018 ^**^	0.006	[0.005, 0.030]	−0.004	0.009	[−0.022, 0.015]	0.020 ^**^	0.006	[0.007, 0.032]
*Gender*												
*Male*	REF.			REF.			REF.			REF.		
*Female*	−0.232	0.167	[−0.559, 0.096]	−0.010	0.118	[−0.242, 0.221]	− 0.225	0.167	[− 0.552, 0.102]	−0.005	0.118	[−0.236, 0.226]
*Health insurance*	−0.408	0.427	[−1.245, 0.428]	−0.778 ^**^	0.286	[−1.338, −0.217]	− 0.461	0.426	[− 1.295, 0.373]	− 0.814 ^**^	0.286	[− 1.374, − 0.254]
*Financial situations*	0.048	0.030	[− 0.010, 0.106]	0.088 ^**^	0.021	[0.047, 0.129]	0.045	0.030	[−0.013, 0.103]	0.084 ^**^	0.021	[0.042, 0.125]
*Chronic conditions*	0.125	0.070	[−0.012, 0.263]	0.146 ^**^	0.047	[0.054, 0.238]	0.124	0.071	[−0.015, 0.262]	0.145 ^**^	0.047	[0.052, 0.238]
*Self-rated health*	−0.045	0.101	[−0.243, 0.153]	−0.143 ^*^	0.064	[−0.269, − 0.017]	−0.046	0.101	[−0.244, 0.151]	− 0.143 ^*^	0.064	[− 0.268, − 0.017]
Intercept	−5.508 ^**^	1.301	[−8.057, −2.959]	−4.679 ^**^	0.951	[−6.542, − 2.815]	− 5.616 ^**^	1.578	[− 8.709, − 2.524]	−6.071 ^**^	1.197	[− 8.416, −3.725]
Num. of non-missing observations	1838						1838					
Wald-statistics	324.13						317.15					
[p-value]	[0.000]						[0.000]					

*p < 0.05, **p < 0.01. Robust standard errors are reported

We also combine the Heckman two-stage procedure and multiple logit model for robustness check, which addresses the concern about potential self-selection bias. Considering the difficulty in instrumental activity of daily living (IADL), the beneficiary status of health insurance, chronic conditions and wealthier financial status may affect the health care service need, it is expected that such pre-determined health care need can influence the class of health care facilities visited. Therefore, in the first-stage, we conduct a regression and obtain the inverse Mills ratio (IMR), and in the second-stage, we adjust the potential self-selection estimation bias by including the IMR into the multiple logit model.

$$Health\ care\ service\ need=\beta 0+\beta 1\ Instrumental\ activity\ of\ daily\ living\ \left[ IADL\right]+\beta 2\ Age+\beta 3\ Gender+\beta 4\ Health\ insurance+\beta 5\ Chronic\ conditions+\beta 6\ Financial\ status+\varepsilon\ \left[1 st\ stage\ regression\right]$$

*Where, the health care service need is measured by “physical examinations” (0-1 indicator, =1 if taken one of the items including physical examination, routine blood test, routine urine test, liver function test, kidney function test, lipids profile test, blood glucose test, surgical, internal medicine, ophthalmology and otorhinolaryngology, electrocardiogram, ultrasonic, chest fluoroscopy, andrology and gynecology; =0 otherwise).*

Results of Table 4 (Model 1) show that the transport cost in health service seeking (ex-post) is associated with the visit to higher tier health care facilities (1.031, *p* < 0.01 for the second tier) and (1.019, *p* < 0.01 for the third tier). Family support (face-to-face) is also associated with the visit to higher tier health care facilities (0.339, *p* < 0.01 for the second tier) and (0.184, *p* < 0.01 for the third tier). Disability remains negatively associated with the tier health care facilities visited (− 1.207, *p* < 0.01 for the second tier) and (− 0.976, *p* < 0.01 for the third tier). Further, the transport cost in health service seeking (ex-post) might alleviate the negative relationship between disability and the tier health care facilities visited (ex-post) (interaction term = 0.408, *p* < 0.05 for the second tier) and (interaction term = 0.409, *p* < 0.01 for the third tier). These results are still robust when we measure the family support by the online connection (Table 4, Model 2).

**Table 4:** Supplementary robustness check: Clarifying potential self-selection bias of transport and health care need (combining Heckman two-stage procedure and multiple logit model)

	Dependent variables: Class of health care facilities visited [multiple logit model, REF = *first tier (community healthcare centers, township hospital, health care service station, village clinic/private clinic)*]											
	*Model 1*						*Model 2*					
	*second tier (specialized hospital, Chinese medicine hospital)*			*third-tier (general hospital)*			*second tier (specialized hospital, Chinese medicine hospital)*			*third-tier (general hospital)*		
	Coef.	Robust S.E.	95% CI	Coef.	Robust S.E.	95% CI	Coef.	Robust S.E.	95% CI	Coef.	Robust S.E.	95% CI
**Independent variables**												
*Transport cost in health service seeking [ex-post, having paid]*	1.031 ^**^	0.090	[0.854, 1.207]	1.019 ^**^	0.074	[0.875, 1.164]	1.036 ^**^	0.090	[0.859, 1.213]	1.030 ^**^	0.075	[0.884, 1.177]
*Family support [face to face]*	0.339 ^**^	0.103	[0.138, 0.541]	0.184 ^**^	0.077	[0.034, 0.335]						
*Family support [Online connection]*							0.354 ^*^	0.138	[0.083, 0.624]	0.328 ^**^	0.104	[0.125, 0.531]
*Disability*	−1.207 ^**^	0.420	[−2.031, −0.384]	−0.976 ^**^	0.308	[−1.579, − 0.373]	− 1.217 ^**^	0.420	[−2.039, − 0.394]	− 0.986 ^**^	0.310	[− 1.594, − 0.378]
*Disability ×Transport cost in health service seeking [ex-post, having paid]*	0.408 ^*^	0.180	[0.055, 0.762]	0.409 ^**^	0.154	[0.106, 0.711]	0.418 ^*^	0.183	[0.060, 0.776]	0.420 ^**^	0.157	[0.112, 0.728]
**Demography**												
*Age*	−0.007	0.014	[− 0.035, 0.021]	0.028 ^**^	0.010	[0.008, 0.047]	−0.008	0.014	[−0.036, 0.020]	0.029 ^**^	0.010	[0.009, 0.050]
*Gender*												
*Male*	REF.			REF.			REF.			REF.		
*Female*	−0.264	0.228	[−0.711, 0.182]	0.166	0.173	[−0.174, 0.505]	−0.269	0.229	[−0.719, 0.181]	0.158	0.176	[−0.188, 0.503]
*Health insurance*	−0.426	0.510	[−1.426, 0.574]	−0.598	0.367	[−1.318, 0.122]	−0.500	0.510	[−1.500, 0.500]	−0.634	0.369	[−1.357, 0.089]
*Financial situations*	0.054	0.064	[−0.072, 0.180]	0.050	0.047	[−0.041, 0.142]	0.058	0.065	[−0.069, 0.185]	0.051	0.048	[−0.042, 0.145]
*Chronic conditions*	0.144	0.079	[−0.011, 0.299]	0.161 ^**^	0.053	[0.057, 0.265]	0.145	0.080	[−0.010, 0.301]	0.165 ^**^	0.054	[0.060, 0.271]
*Self-rated health*	−0.104	0.128	[−0.355, 0.147]	−0.148	0.082	[−0.308, 0.013]	− 0.107	0.128	[− 0.357, 0.143]	−0.148	0.082	[−0.309, 0.012]
*Inverse Mills ratio (adjusted according to health care need)*	65.104	175.500	[− 278.871, 409.078]	−100.521	136.470	[−367.996, 166.954]	87.438	178.278	[− 261.981, 436.857]	−83.035	141.805	[− 360.967, 194.896]
Intercept	−23.929	50.083	[−122.091, 74.232]	24.282	38.968	[−52.093, 100.657]	−30.500	50.938	[− 130.338, 69.337]	17.709	40.466	[−61.604, 97.022]
Num. of non-missing observations	1373						1373					
Wald-statistics	282.18						272.89					
[p-value]	[0.000]						[0.000]					

^*^p < 0.05, ^**^p < 0.01. Robust standard errors are reported

## Discussion

### General discussion

Based on social ecological modeling, this study investigates the health services seeking behavior of older people with and without disabilities by integrating different determinants. Family support (e.g., care and emotional comfort) could increase the importance that older people attach to their own health [49]. Regular contact and care could alleviate the inability and perceived difficulty of older people with disabilities in seeking health care services.

The study found that transport cost can be an important factor of access to advanced health care facilities, such that people who seek advanced health care services would have to pay higher transport cost, which is consistent with previous research findings [50]. In general, high-level health care institutions can provide more comprehensive services. However, high-level health care institutions are mainly located in the core region with dense population, due to the functional integrity of health care resources. In contrast, lower-level health care facilities (e.g., community health service centers, township health centers and health service stations) are distributed in communities, and it often does not take much time to reach the basic health care service. In addition to the external environment, disability as a personal characteristic is also found to be negatively associated with access to higher-class health care facilities. Older people with limitations and deficiencies in activities impair their ability to access to health care services [51], which is consistent with previous research finding that people with disabilities in rural areas face more barriers to access to health care than counterparts, especially in the context of LMICs [52].

### Policy implication

This study has some important policy implications, and shed light on the influence mechanism that affects health care service of older people with disabilities. First, it is necessary for policymakers to expand the coverage of the transport network to reduce the cost of travel distance. Increase in the frequency and routes of public transportation can optimize the travel distance to health care facilities for older people living in rural or suburban areas. Second, the establishment of special transportation lines for older people with disabilities can also be considered to help them to reach medical facilities and promote the health equity of accessing medical resources. Third, family members should continue to play a role in influencing older people’s health behaviors. Family members should also enhance their care for older family members by strengthening the connection within the family. In the context of LMICs where resource constraint can be severe, the fulfillment of economic support and psychological attention from family can be both important.

## Conclusion

This study shows that alleviating transport deprivation and facilitating family support are critical for access to better health services among older people especially those with disabilities in the context of LMICs.

## Data Availability

The data are publicly available upon reasonable request and via online application to the CHARLS research team.

## Abbreviations

CHARLS: China Health and Retirement Longitudinal Study
HHCF: Higher-class health care facilities
IADL: Instrumental activity of daily living
LMICs: Low- and middle-income countries
